# Factors associated with quality of life in systemic sclerosis: a cross-sectional study

**DOI:** 10.1007/s11136-019-02284-9

**Published:** 2019-09-03

**Authors:** Matylda Sierakowska, Halina Doroszkiewicz, Justyna Sierakowska, Marzena Olesińska, Agnieszka Grabowska-Jodkowska, Marek Brzosko, Piotr Leszczyński, Katarzyna Pawlak-Buś, Bogdan Batko, Piotr Wiland, Maria Majdan, Małgorzata Bykowska-Sochacka, Wojciech Romanowski, Aleksandra Zon-Giebel, Sławomir Jeka, Mwidimi Ndosi

**Affiliations:** 1 Department of Integrated Medical Care, Medical University of Bialystok, 7a Maria Sklodowska-Curie Street, 15-096 Bialystok, Poland; 2 Department of Geriatrics, Medical University of Bialystok, Bialystok, Poland; 3 Department of Foreign Languages, Medical University of Bialystok, Bialystok, Poland; 4 Department of Connective Tissue Disease, National Institute of Geriatrics, Rheumatology and Rehabilitation, Warsaw, Poland; 5 Department of Rheumatology, Internal Diseases and Geriatrics, Pomeranian Medical University in Szczecin, Szczecin, Poland; 6 Department of Rheumatology and Rehabilitation, Medical University in Poznań, Poznań, Poland; 7 Center of Rheumatology, J. Dietl Hospital in Krakow, Krakow, Poland; 8 Department of Rheumatology and Internal Diseases and Geriatrics, Medical University in Wroclaw, Wroclaw, Poland; 9 Department of Rheumatology and Connective Tissue Diseases, Medical University in Lublin, Lublin, Poland; 10 Dr J. Titz-Kosko Regional Hospital for Rheumatic Diseases, Sopot, Poland; 11 Poznań Centre of Rheumatology, Śrem, Poland; 12 Silesian Center of Rheumatology, Rehabilitation and Prevention of Disability, Ustroń, Poland; 13 Department of Rheumatology and Connective Tissue Diseases, 2nd University Hospital in Bydgoszcz, Bydgoszcz, Poland; 14 Department of Nursing and Midwifery, University of the West of England, Bristol, UK

**Keywords:** Systemic sclerosis, Quality of life, Physical disability, Anxiety

## Abstract

**Introduction:**

Systemic sclerosis (SSc) is a connective tissue disease characterized by progressive fibrosis of the skin and internal organs, leading to their failure and disturbances in the morphology and function of blood vessels. The disease affects people in different ways, and identifying how the difficulties and limitations are related to quality of life may contribute to designing helpful interventions. The aim of this study was to identify factors associated with quality of life in people with SSc.

**Methods:**

This was a cross-sectional study conducted in 11 rheumatic centres in Poland. Patients diagnosed with SSc were included. Quality of life was measured using the SSc Quality of Life Questionnaire (SScQoL). The following candidate factors were entered in preliminary multivariable analysis: age, place of residence, marital status, occupational status, disease type, disease duration, pain, fatigue, intestinal problems, breathing problems, Raynaud’s symptoms, finger ulcerations, disease severity, functional disability, anxiety and depression. Factors that achieved statistical significance at the 10% level were then entered into a final multivariable model. Factors achieving statistical significance at the 5% level in the final model were considered to be associated with quality of life in SSc.

**Results:**

In total, 231 participants were included. Mean age (SD) was 55.82 (12.55) years, disease duration 8.39 (8.18) years and 198 (85.7%) were women. Factors associated with quality of life in SSc were functional disability (*β* = 2.854, *p* < 0.001) and anxiety (*β* = 0.404, *p* < 0.001). This model with two factors (functional disability and anxiety) explained 56.7% of the variance in patients with diffuse SSc and 73.2% in those with localized SSc.

**Conclusions:**

Functional disability and anxiety are significantly associated with quality of life in SSc. Interventions aimed at improving either of these factors may contribute towards improving the quality of life of people with SSc.

**Electronic supplementary material:**

The online version of this article (10.1007/s11136-019-02284-9) contains supplementary material, which is available to authorized users.

## Introduction

Rheumatic diseases, especially chronic non-infectious arthritis and the so-called systemic connective tissue diseases, hold a special place among chronic diseases; they are the main cause of organ dysfunction, disability and even disability or premature death [1, 2]. Over the last few decades, there has been great progress in improving the effectiveness of their treatment. At the turn of the century, biological drugs have allowed not only to slow down, but, for the first time in history, stop the destruction of joints which is the essence of inflammatory rheumatic diseases [3]. The exception to this is systemic sclerosis (SSc), which is a chronic disease with autoimmune background.

SSc is characterized by progressive fibrosis of the skin and internal organs, leading to their failure and disturbances of morphology and function of blood vessels [4]. SSc is not a uniform disease. There are several clinical forms, including mainly limited systemic sclerosis (lSSc) and diffuse systemic sclerosis (dSSc) [2, 3]. SSc is a rare condition with global incidence estimated to be between 3 and 24 per 100,000 population. It is higher in North America and Australia compared to Europe and Japan [5]. About 10,000 people live with SSc in Poland (1 patient in about 4000 people from the entire population). Women are 3 to 5 times more likely than men to have SSc. Disease incidence is highest between 35 and 55 years [2, 3].

Currently, there is no curative treatment or drugs that effectively inhibit or at least significantly delay the progression of the disease. However, in the last few decades, the survival rate of patients with SSc has been somewhat extended. This is the result of the use of organ-specific therapy, based on the assumptions of the complexity of therapeutic treatment and the individualization of treatment, taking into account the duration of the disease and the advancement of organ changes [6].

As a chronic disease, SSc affects patients’ quality of life in many ways. Some of the problems include feeling tired; limitation of the ability to perform everyday activities, especially manual ones; unpredictable course of the disease, especially the dSSc form; distress related to skin and internal organs ailments; sleep disorders; unacceptance of change of look of face and general appearance, low self-esteem; deficit of knowledge about the disease, leading to ineffective self-care [7–10].

Health-Related Quality of Life (HRQoL) is defined as the influence of the disease and treatment on functioning and overall sense of life satisfaction perceived by the patient [11]. Improving patients’ quality of life is the mark of a holistic approach to address the complex problems of the patient with chronic diseases such as reduced tolerance of treatment, ineffective therapies or risks associated with co-morbid diseases. This helps clinicians, nurses and health professionals to identify areas that are amenable to change in order to improve the patient’s quality of life, alongside medical goals [7]. The aim of this study was to determine factors associated with quality of life in people with SSc.

## Methods

### Design

This was a multicentre cross-sectional analytical study carried out in 11 rheumatology centres in Poland. Patients’ inclusion criteria were a diagnosis of SSc, based on ACR/EULAR 2013 classification [12] and ages of 18 years and above. The exclusion criterion was the coexistence of another known systemic connective tissue disease.

### Ethics consideration

The research was carried out in accordance with the Declaration of Helsinki and Good Clinical Practice in research. The Bioethics Committee of the Medical University in Bialystok, Poland granted the ethical approval for the study (R-I-002/175/2016). Participation was voluntary and participants were informed about the project and gave written consent.

### Measures

To measure quality of life (outcome variable), we used a disease-specific measure, the *Systemic Sclerosis Quality of Life Questionnaire* (*SScQoL*), developed using a needs-based quality of life model which was validated for Poland and 7 other European countries using Rasch analysis [13]. The tool has 29 questions divided into five subscales relating to physical functioning (score range: 0 to 6), emotional functioning (0 to 13), social functioning (0 to 6), sleep (0 to 2) and pain (0 to 2). High scores indicate a greater impact of the disease, i.e. decrease of HRQoL [14].

We expected the following factors (explanatory variables) to be associated with quality of life in SSc as they represent some aspects of disease impact: functional disability (patients’ difficulty in performing daily activities), pain, fatigue, intestinal problems, breathing problems, Raynaud’s symptoms, finger ulcerations, patient’s own assessment of disease severity and psychological distress. Personal factors (age, type of SSc, disease duration, place of residence, marital status and occupational status) were also considered in addition to the above, making a total of 16 candidate factors.

Functional disability was assessed using the *Health Assessment Questionnaire-Disability Index* (*HAQ*-*DI*), which comprises 20 items divided into 8 sections: dressing and washing, morning rising, eating, walking, personal hygiene, lifting, grasping and movement. The total score ranges from 0 to 3: the higher the score, the worse the level of physical functioning [14, 15]. Based on the *Visual Analogue Scale* (*VAS 0 to 100*), the following health problems were assessed: pain intensity (pain-VAS), fatigue (fatigue-VAS), intestinal problems, breathing problems, severity of Raynaud’s symptoms, severity of finger ulcerations and general evaluation of the disease severity [16]. Psychological distress (anxiety and depression) was assessed using the *Hospital Anxiety and Depression Scale* (*HADS*). The questionnaire consists of two subscales: anxiety (HADS-A, *0 to 21*) and depression (HADS-D, *0 to 21*), each containing 7 statements. A score of 0 to 7 in each subscale means the norm, no disorders; a score of 8 to 10 means suspicion of anxiety and/or depressive disorders; score of 11 or higher indicates the possibility of anxiety or depression disorders, respectively [17]. The participants completed the questionnaires unaided. While the SScQoL measures the impact of disease on health and well-being, all the above factors measure different aspects of disease impact but not comprehensively. They were therefore expected to correlate with the SScQoL, and in our analyses below, we entered all these candidate factors into a multivariable model to test which ones would independently be associated with SScQoL.

### Sample size estimation

As SSc is a rare condition, 11 centres were recruited and were asked to invite all eligible patients allowing a 30% response rate. We recruited 231 patients and tested the 16 prespecified candidate factors in a multiple linear regression. Post hoc power analysis showed that a sample size of 226 would achieve power of 97% when including 16 explanatory variables and an alpha level of 0.05.

### Data analysis

The raw scores from the SScQoL which are ordinal in nature were transformed into interval level measures using calibrated conversion tables (online supplementary material 1) before being combined with other data and subjected to parametric analyses. All data were then summarized descriptively using means and standard deviations. To assess differences between subgroups of patients with lSSc and dSSc, univariable analyses were conducted. To identify factors associated with quality of life in SSc, all 16 prespecified factors (age, type of SSc, disease duration, place of residence, marital status, occupational status, pain, fatigue, intestinal problems, breathing problems, Raynaud’s symptoms, finger ulcerations, disease severity, functional disability, anxiety and depression) were entered in a preliminary multivariable analysis. Factors that achieved statistical significance at the 10% level were then entered into a final multivariable model. For these analyses, we present parameter estimates (β) with corresponding 95% CIs and *p* values for factors found to be significantly associated with quality of life in SSc at the 0.05 alpha level. All data were analysed using PQStat v.1.4.2 software.

## Results

### The characteristics of patients with SSc

In total, 231 patients with SSc participated in the study, 114 (49.4%) had lSSc and 117 (50.6%) had dSSc. Average (SD) age of the participants was 55.82 (12.55) years, disease duration 8.39 (8.18) years and the majority were women 198 (85.7%). The majority, 160 (69.3%), of the patients were in a relationship/married and 151 (65.4%) were on disability pension/retirement. The characteristics of patients are summarized in Table 1.

**Table 1:** Characteristics of patients with SSc

Variables studied (score range)	*n* (%)	Mean (SD)
Age in years		55.82 (12.55)
Gender—number of women	198 (85.7)	
Educational background		
Basic	22 (9.5)	
Professional	61 (26.4)	
Secondary	95 (41.1)	
Higher education	53 (22.9)	
Place of residence		
Urban (> 100,000)	85 (36.8)	
Urban (up to 100,000)	72 (31.2)	
Rural	74 (32.0)	
Marital status		
In a relationship/married	160 (69.3)	
Single	71 (30.7)	
Occupational status		
High school student/college student	7 (3.0)	
Employed	57 (24.7)	
Unemployed	16 (6.9)	
Person on the disability allowance/retired	151 (65.4)	
Clinical form systemic sclerosis (SSc)		
lSSc	114 (49.4)	
dSSc	117 (50.6)	
Disease duration in years		8.39 (8.18)

*lSSc* limited systemic sclerosis, *dSSc* diffuse systemic sclerosis

### Differences between patients with dSSc and lSSc

Table 2 presents differences in health problems and quality of life between the participants with dSSc and those with lSSc. Participants with dSSc reported higher levels of functional disability, pain, fatigue and self-reported disease severity than those with lSSc. Significant differences were also observed in relation to the occurrence of finger ulceration and breathing problems. Patients with dSSc had worse quality of life than those with lSSc.

**Table 2:** Difference in health problems and quality of life between patients with dSSc and lSSc

Variables studied (score range)	Total patients *n* = 231	Clinical form of SSc		Difference between groups
		lSSc *n* = 114	dSSc *n* = 117	
	Mean (SD)	Mean (SD)	Mean (SD)	*F* statistic (*p* value)
HAQ-DI (0–3)	0.99 (0.75)	0.83 (0.73)	1.15 (0.74)	11.06 **(0**.**001)**
Pain-VAS (0–100)	33.08 (28.36)	29.26 (27.86)	36.80 (28.46)	4.14 **(0**.**043)**
Fatigue-VAS (0–100)	43.00 (29.04)	38.56 (30.20)	47.33 (27.30)	5.37 **(0**.**021)**
Disease severity (0–100)	41.32 (27.10)	35.79 (28,41)	46.71 (24.71)	9.73 **(0**.**002)**
Raynaud’s attacks (0–100)	38.10 (31.90)	34.12 (31.51)	41.97 (31.94)	3.53 (0.062)
Intestinal problems (0–100)	20.18 (27.73)	17.68 (25.60)	22.62 (29.55)	1.85 (0.186)
Breathing problems (0–100)	26.57 (27.59)	22.32 (26.38)	30.71 (28.22)	5.44 **(0**.**021)**
Finger ulceration (0–100)	17.50 (27.85)	12.94 (25.07)	22.03 (29.77)	6.24 **(0**.**013)**
HADS-A (0–21)	8.96 (4.67)	8.68 (5.03)	9.22 (4.28)	0.77 (0.382)
HADS-D (0–21)	6.70 (4.12)	6.44 (4.47)	6.96 (3.74)	0.92 (0.340)
SscQoL (0–29)	14.81 (5.08)	13.89 (5.56)	15.71 (4.40)	7.58 **(0**.**006)**
SscQoL (domains)				
Physical functioning (0–6)	3.66 (1.57)	3.40 (1.59)	3.91 (1.51)	6.29 **(0.013)**
Emotional functioning (0–13)	6.43 (3.04)	6.03 (3.27)	6.82 (2.75)	3.94 **(0.048)**
Social functioning (0–6)	2.25 (1.87)	1.84 (1.79)	2.65 (1.85)	11.59 **(0**.**001)**
Sleep (0–2)	0.96 (0.91)	0.85 (0.92)	1.07 (0.89)	3.33 (0.069)
Pain (0–2)	0.98 (0.84)	0.82 (0.83)	1.14 (0.82)	8.25 **(0**.**004)**

Bold value indicates the level of significance* p* ≤ 0.05

*lSSc* limited systemic sclerosis, *dSSc* diffuse systemic sclerosis, *HAQ-DI* Health Assessment Questionnaire-Disability Index, *VAS* Visual Analogue Scale, *SScQoL* Systemic Sclerosis Quality of Life Questionnaire

### Factors associated with quality of life in people with SSc

Table 3 presents the results of the multivariable analyses. After fitting all candidate factors into a preliminary multivariable model, four factors were statistically significant at 10% level. These were functional disability (*β* = 2.939, *p* < 0.001), fatigue severity (*β* = 0.020, *p* = 0.036), disease severity (*β* = 0.017, *p* = 0.096) and anxiety (*β* = 0.428, *p* < 0.001). When fitting these four factors in the final multivariable model, only two factors remained statistically significant at 5% level. These were functional disability (*β* = 2.854, *p* < 0.001) and anxiety (*β* = 0.404, *p* < 0.001). The results suggest that a model with two factors (functional disability and anxiety) explained 56.7% of the variance in dSSc and 73.2% in lSSc and was a significant factor associated with quality of life in people with the two clinical forms of SSc.

**Table 3:** Factors associated with quality of life in SSc—results of multivariable analyses

Variables	Preliminary multivariable analysis			Final multivariable analysis		
	Parameter estimate (factor β)	95% CI	*p* value	Parameter estimate (factor β)	95% CI	*p* value
Age in years	− 0.006	− 0.037 to 0.024	0.683			
Type of SSc	0.165	− 0.494 to 0.825	0.622			
Disease duration in years	0.012	− 0.029 to 0.054	0.565			
Place of residence	0.285	− 0.110 to 0.680	0.156			
Family situation	0.149	− 0.119 to 0.418	0.274			
Occupational status	− 0.048	− 0.460 to 0.364	0.818			
HAQ-DI	2.939	2.272 to 3.605	**< 0.001**	2.854	5.745 to 3.403	**< 0.001**
Pain	− 0.001	− 0.022 to 0.019	0.895			
Fatigue	0.020	0.001 to 0.040	**0.036**	0.014	− 0.002 to 0.031	0.082
Disease Severity	0.017	− 0.003 to 0.038	**0.096**	0.016	− 0.001 to 0.033	0.070
Raynaud’s attacks	− 0.008	− 0.022 to 0.005	0.225			
Intestinal problems	− 0.006	− 0.021 to 0.008	0.400			
Breathing problems	0.006	− 0.010 to 0.022	0.444			
Finger ulceration	0.003	− 0.011 to 0.017	0.690			
HADS-A	0.428	0.313 to 0.543	**< 0.001**	0.404	0.326 to 0.482	**< 0.001**
HADS-D	− 0.009	− 0.145 to 0.127	0.896			

*HAQ-DI* Health Assessment Questionnaire-Disability Index, *HADS-A* Hospital Anxiety and Depression Scale-Anxiety subscale, *HADS-D* Hospital Anxiety and Depression Scale-Depression subscale

Bold variables denote significant factors associated with quality of life in SSc

## Discussion

To our knowledge, this is the first time the SScQoL has been used to assess quality of life of people with SSc in a large population. A smaller study recently conducted in Sweden (*n* = 67) [18] showed that the SScQoL had a better ability to capture the disease-specific factors influencing quality of life in SSc than the generic measure of quality of life EQ 5D. Cross-cultural validation of the SScQoL into Polish was part of an international collaboration [13] and this allows the measure to be used in multinational research and cross-cultural comparisons. The key findings of our study are that functional disability (limitations of physical function) and anxiety are significantly associated with quality of life in people with SSc (dSSc and lSSc). These two factors are amenable to change and therefore can be a target for non-pharmacological interventions by health professionals.

The above results are supported by previous studies. An Italian study by Danieli et al. [9] using SF36 showed that limitations in the ability to perform daily activities were the main reason for poor HRQoL in patients with SSc [9]. According to Sandqvist et al., [19] the difficulties in performing activities involve mainly manual abilities such as eating, drinking, washing and dressing. Other studies indicate that the symptom of “tight glove” characteristic for SSc (painful cracks and sores of the fingers, making it difficult to perform precise tasks) may limit the professional and social activity of patients, causing stress and as a result lowering the quality of life [20, 21]. Non-pharmacological interventions by occupational therapists may help address some aspects of functional disability, by providing adaptations and skills to help patients to be independent and live as normal a life as possible. Our findings also suggest that anxiety is a significant factor associated with quality of life in SSc. Anxiety disorders are a significant problem for people with SSc (affecting over 80% of participants in our study) and correlate positively with depression [22]. In our study, almost half of the participants had HADS-A scores suggesting a suspected or moderate anxiety disorder, and more than a third suspected or moderate severity of depressive disorders. Anxiety has been shown to be associated with other long-term conditions such as diabetes and rheumatoid arthritis. Whether the anxiety in SSc is generalized or disease specific, interventions to address anxiety are likely to help improve quality of life in people with SSc.

Analysis of the results by clinical presentations showed that health problems (ulceration of the fingers, disability, dyspnoea, fatigue, pain) were more severe in the dSSc and consequently quality of life in patients with dSSc was worse than those with lSSc. While this finding is interesting, disease type in the multivariable analyses was not shown to be an independent factor associated with quality of life. This suggests that interventions to improve quality of life should be targeted to both disease types equally.

Although other factors were not shown to be significantly associated with quality of life in SSc, it is important to note that they may pose problems in the lives of people with SSc. One of these factors is finger (digital) ulcers which are very painful, difficult to treat and severely limit activities of daily living. Although very few patients with finger ulceration were represented, these pose a significant burden on people with SSc. Fatigue also is a common problem in people with rheumatic diseases, having a physical, psychological as well as a social dimension [7, 8]. Fatigue can be a significant cause of suffering and disability in people with SSc [23]. There is a complex association between fatigue, pain and physical functioning. Pain has been shown to be strongly associated with fatigue, depression and diminished physical functioning [21, 24–31]. Previous studies have shown that pain, finger ulcerations, Raynaud’s symptoms and the involvement of internal organs, especially the lungs, make it difficult to perform everyday tasks and may also limit social functioning [8, 29–31]. The severity of the disease may be associated with the risk of emotional problems [8] and this has been evidenced in our study. Depression affects the physical as well as the psychological quality of life [32, 33]. Benrud-Larson et al., [34] reported that organ changes in the course of SSc, as well as physical limitations, hinder social adaptation and may predispose patients to depression. Also, shiny, tense, thickened skin, telangiectasias and microstomia cause aesthetic changes to the face, which causes dissatisfaction, reduced self-esteem, deepening depression and limited psycho-social functioning of people with SSc [35, 36]. Family relationships and place of residence may also play a role in patients’ quality of life. For example, people with SSc who are also caring for their partner or loved ones may have additional responsibilities that may affect their self-care and quality of life.

The interest in addressing quality of life in people with scleroderma emanates from a holistic approach to care for patients with chronic, inflammatory and connective tissue diseases, which is at the heart of rheumatology nursing. Self-reported HRQoL informs about disease impact and how the patient is coping with new challenges and problems that affect health, social status or psychological functioning [1, 37]. Monitoring of quality of life is an important element of managing the condition and may help to inform appropriate interventions, which are likely to be non-pharmacological and delivered by the multidisciplinary team [38–40].

Our research has two key limitations. First, the participants in the study were those who were attending a medical facility, meaning that selection bias cannot be excluded. However, as SSc is a rare condition and this study was conducted in 11 sites in Poland, including 231 participants (approx. 10,000 people with the condition) provides fairly generalizable results for the population with the condition in Poland [2, 3]. Second, the study was cross-sectional, therefore no causality can be ascribed to the identified factors associated with quality of life in SSc. However, identifying these factors is important as it may help direct the design of important interventions.

## Conclusion

Our study has identified functional disability and anxiety to be the main factors associated with quality of life in people with SSc. The overall aim of disease management is to improve quality of life in people with SSc. Identification of these factors will help design appropriate non-pharmacological interventions, delivered by a multidisciplinary team. Our results also demonstrate that SScQoL captures important disease-specific factors associated with quality of life in SSc and is therefore a useful tool for clinical and research use.

## Electronic supplementary material

Below is the link to the electronic supplementary material.

Supplementary material 1 (PDF 60 kb)
