# Supplementary material for: Factors associated with quality of life in systemic sclerosis: a cross-sectional study
Source: Qual Life Res. 2019 Sep 3;28(12):3347–54. doi: 10.1007/s11136-019-02284-9 (PMC6863937; doi:10.1007/s11136-019-02284-9)
Supplement: Supplementary file 1 — Supplementary material 1 (PDF 60 kb) [file 11136_2019_2284_MOESM1_ESM.pdf]

Supplementary Material 1. **Conversion table for SScQoL raw-to-linear (Rasch transformed) scores**

Raw scores (yes=1, no=0)	Function	Emotional	Sleep	Social	Pain	Total
**0.0**	0.0	0.0	0.0	0.0	0.0	0.0
**1.0**	1.3	1.6	1.0	1.6	1.0	3.6
**2.0**	2.4	2.8	2.0	2.6	2.0	6.0
**3.0**	3.1	3.7		3.3		7.5
**4.0**	3.8	4.5		3.9		8.6
**5.0**	4.8	5.2		4.7		9.6
**6.0**	6.0	5.9		6.0		10.3
**7.0**		6.6				11.0
**8.0**		7.3				11.6
**9.0**		8.0				12.2
**10.0**		8.9				12.8
**11.0**		9.9				13.3
**12.0**		11.2				13.8
**13.0**		13.0				14.3
**14.0**						14.8
**15.0**						15.3
**16.0**						15.8
**17.0**						16.3
**18.0**						16.8
**19.0**						17.3
**20.0**						17.8
**21.0**						18.4
**22.0**						19.0
**23.0**						19.7
**24.0**						20.4
**25.0**						21.3
**26.0**						22.4
**27.0**						23.8
**28.0**						25.9
**29.0**						29.0

The SScQoL has dichotomous yes/no responses, coded as 1 (yes) and 0 (no), yielding a scoring range 0–6 for the function subscale, 0–13 for the emotional subscale, 0-2 for sleep and so on. The scores obtained from the patient are the raw scores and these must be converted to linear scores using the conversion chart. For example, if a patient has a raw score of 2 on the emotional subscale, this will be transformed to 2.8, if the patient has a raw score of 3 on the function subscale this will transformed to 3.1, and so on in the other subscales. Adding up all the transformed subscale scores gives the total SScQoL score which is an estimate of the patient's quality of life (range 0–29), higher scores indicating a worse quality of life.
